# Supplementary material for: Histopathological domain adaptation with generative adversarial networks: Bridging the domain gap between thyroid cancer histopathology datasets
Source: PLoS One. 2024 Dec 26;19(12):e0310417. doi: 10.1371/journal.pone.0310417 (PMC11670965; doi:10.1371/journal.pone.0310417)

**S1. Additional information regarding subtype classifications**

**Follicular cell-derived neoplasms.** Benign thyroid tumours can be classified into several types based on their histopathological features. The most common types of benign thyroid tumours include:

- Follicular adenoma (FA): FA is derived from follicular cells of the thyroid gland. It is characterized by the presence of well-defined follicles that are lined by a single layer of cuboidal epithelial cells. These tumours are usually encapsulated and can be difficult to distinguish from normal thyroid tissue [59].
- Follicular adenoma with papillary architecture: This tumour is usually encapsulated with intra-follicular centripetal papillary growth, and the lesional cells lack nuclear features of papillary thyroid carcinoma (PTC) [60].
- Oncocytic adenoma of the thyroid: Both benign and malignant oncocytic thyroid tumours exhibit papillary architecture and are composed of oncocytes, which are large, eosinophilic cells with granular eosinophilic cytoplasm [61].

Overall, benign thyroid tumours are usually well-circumscribed and show a relatively uniform population of cells with no evidence of invasion or metastasis. However, it is important to note that some benign tumours can have atypical features that may require careful monitoring or even surgical removal. It is worth noting that although these tumours are generally considered benign, there is always a risk that they could progress to malignancy.

**Malignant thyroid tumours.**

- **Papillary thyroid carcinoma (PTC):** PTC is the most common type of thyroid cancer, accounting for approximately 80% of cases. It arises from the follicular cells of the thyroid gland [62].

The histopathology of PTC typically shows well-differentiated papillary structures with a fibrovascular core (hallmark of PTC). These structures are lined by one or several layers of neoplastic epithelial cells with crowded nuclei. The nuclei may display irregular contours, nuclear pseudo-inclusions and nuclear membrane irregularities (nuclear grooves). The chromatin is characterised by chromatin clearing, margination and glassy nuclei [31, 60, 63].

In addition, PTC may exhibit psammoma bodies, which are concentrically laminated calcific nodules that represent the ghosts of dead papillae. These are a characteristic finding in PTC [63].

In advanced cases, the cancerous cells can invade through the thyroid capsule, blood vessels, and lymphatics. Overall, the histopathology of PTC is characterized by well-differentiated papillary structures with neoplastic epithelial cells displaying nuclear changes that are considered a diagnostic hallmark of this tumour entity and this is shown in the World Health Organization (WHO) classification [64].

- **Papillary thyroid carcinoma-like tumours (PTC-like):** PTC-like tumours are a group of neoplasms that are histologically similar to PTC but are not true PTCs. They are considered a type of follicular cell-derived thyroid neoplasm. These tumours are typically characterized by the presence of papillary projections and nuclear features that are closely resembling PTC. However, unlike PTC, these tumours do not have the characteristic growth pattern and are not confined to the thyroid gland.

Some examples of PTC-like tumours include:

- - **Non-invasive follicular thyroid neoplasm with papillary-like nuclear features (NIFTP):** NIFTP is a non- invasive tumour that was previously classified as a variant of PTC. However, it is now recognized as a distinct entity by the WHO [64].

NIFTP is diagnosed based on specific histopathological features which include the presence of follicular architecture, absence of invasion, and nuclear features resembling those seen in PTC but less prominent [65].

NIFTP is considered to have a very low risk of recurrence or progression to malignancy and is therefore classified as a low-risk neoplasm [64]. It is important to distinguish NIFTP from PTC which have a higher risk of recurrence and require more aggressive treatment.

- **Follicular variant of papillary thyroid carcinoma (FV-PTC):** FV-PTC is a subtype of PTC. The histopathology of FV-PTC is characterized by the presence of follicular architecture and nuclear features typical to PTC. In addition to the typical nuclear features, FV-PTC may also show areas of vascular invasion and perineural invasion, which are associated with a higher risk of recurrence and metastasis [60, 64].

The tumour is usually well-circumscribed and encapsulated [60].

A definitive diagnosis of FV-PTC requires the presence of nuclear features of PTC in the follicular architecture.

- **Nonencapsulated sclerosing carcinoma or diffuse sclerosing variant of papillary thyroid carcinoma (DSV-PTC):** A rare subtype of PTC, accounting for less than 1% of all thyroid malignancies. It is characterized by diffuse infiltration of the thyroid gland by tumour cells, resulting in a diffusely enlarged gland with no discrete nodules.

Histologically, DSV-PTC is characterized by the presence of a sclerotic stroma, which is composed of fibrous tissue and lymphocytes. The tumour cells are arranged in a diffuse and infiltrative pattern, with no well-defined follicles or papillae. The nuclei of the tumour cells exhibit nuclear features of PTC. Psammoma bodies may be present within the tumour [66].

DSV-PTC is often associated with lymph node metastases and a higher incidence of extrathyroidal extension compared to other types of PTC. It has also been reported to have a more aggressive clinical course and a higher recurrence rate, although this is still a matter of debate [66].

In summary, PTC-like tumours are a group of neoplasms that are histologically similar to PTC but have different growth patterns and clinical characteristics. The treatment and prognosis of these tumours may vary depending on the specific subtype.

- **Follicular thyroid carcinoma (FTC):** is the second most common type of thyroid cancer, accounting for approximately 10-15% of all cases. FTC is derived from the follicular cells of the thyroid gland [67, 68].

FTC shows a characteristic pattern of follicular growth, lined with neoplastic cells without the nuclear features of PTC [68]. The histopathology of FTC is indistinguishable from follicular adenoma (FA) but the former appears more cellular and with irregular thick capsule [59].

Histologically, FTC is distinguished from FA by the presence of capsular or vascular invasion. The presence of invasion is usually confirmed by examining the tumour capsule or the surrounding thyroid tissue for the presence of tumour cells. If invasion is identified, the diagnosis of FTC is made. In addition, FTC may show vascular invasion, with tumour cells present within blood vessels. This is another important diagnostic criterion for malignancy [59].

Overall, the histopathology of FTC is characterized by the presence of follicular growth patterns, uniform neoplastic follicular cells, and invasion of the fibrous capsule and/or blood vessels being the hallmark feature that distinguishes it from FA.

- **Oncocytic carcinoma of the thyroid:** The tumour cells are identical to the benign counterpart. The tumour cells may exhibit the nuclear features of PTC or may have pleomorphic and hyperchromatic nuclei. Oncocytic carcinoma is associated with aggressive clinical behaviour in comparison to PTC [61, 69].

**WHO Classification (2022) - Follicular cell–derived neoplasms**

Supplementary material, Figure S1 contains a table of the WHO (2022) designation of the following PTC-like and non-PTC-like subclasses:

- **PTC-like:** NIFTP, PTC, FV-PTC, DSV-PTC
- **Non-PTC-like:** Benign, FTC, Oncocytic/Oxyphilic carcinoma of the thyroid

**Supplementary Material, Figure S1**. WHO (2022) designation of subclasses which appear in the main report.


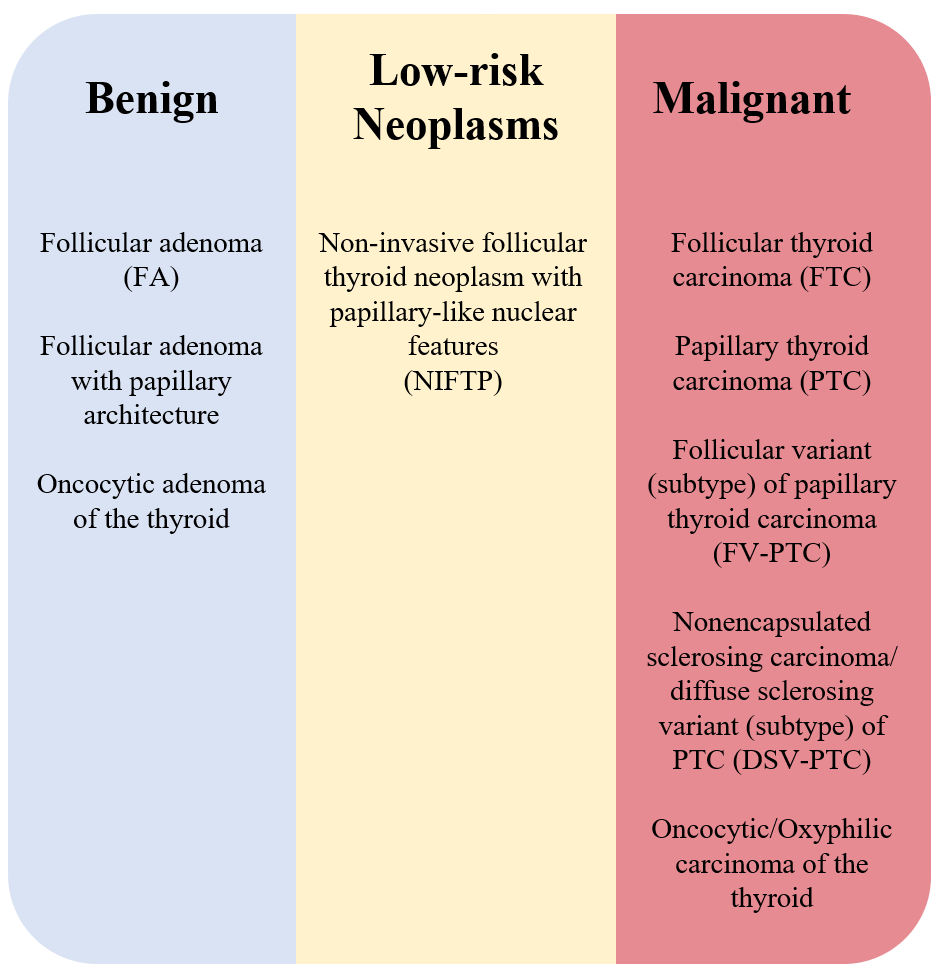

Supplement: S1 File — Detailed information pertaining to the Thyroid tumour classification subtypes which are relevant to this report, including WHO 2022 designations. (DOCX) [file pone.0310417.s001.docx]
