# Supplementary material for: Histopathological domain adaptation with generative adversarial networks: Bridging the domain gap between thyroid cancer histopathology datasets
Source: PLoS One. 2024 Dec 26;19(12):e0310417. doi: 10.1371/journal.pone.0310417 (PMC11670965; doi:10.1371/journal.pone.0310417)
Supplement: S2 File — Additional details about the selection of the samples included within the NTE dataset. (DOCX) [file pone.0310417.s002.docx]

**S2. NTE dataset selection**

Included below is any additional information required to replicate the construction of the NTE dataset which is not included within the main report (see Methods: Data acquisition and processing).

***Nikiforov Data.*** The “Nikiforov” dataset used by [20] consisted of 138 patient samples (103 PTC-like, 30 non-PTC-like) taken from “BoxA” of the Nikiforov online repository. The labels of these samples are neither shared by the reference paper nor are in the repository.

Therefore, the designation of PTC-like or non-PTC-like could only be determined for 55 samples which were explicitly scored within Tables 5 and 6 of the Nikiforov supplementary materials [31]. Of these, 36 samples remain uploaded in BoxA. 25 are designated as NIFTP (PTC-like) and 11 are benign (non-PTC-like). These were included to form part of the external dataset used by this paper for testing model generalizability.

***TCGA Data.*** Additional thyroid histopathology samples were sourced from The Cancer Genome Atlas (TCGA) Thyroid Carcinoma study.

The study contains the following samples:

- Papillary adenocarcinoma (PA) – 356 patients
- Follicular variant of papillary thyroid carcinoma (FVPTC) – 105 patients
- Papillary carcinoma, columnar cell (PCC) – 38 patients
- Nonencapsulated sclerosing carcinoma (NSC) – 4 patients
- One patient sample of: follicular thyroid carcinoma (FTC), follicular adenocarcinoma (FAC), and oxyphilic adenocarcinoma (OA)

From this, 30 FVPTC samples were selected as PTC-like samples for the NTE dataset. This ensured a similar number of both minority positive classes (NIFTP and FVPTC) in the T&T training data would be present in the external test data. All non- PTC-like patient samples from TCGA were downloaded, however only three files were uncorrupted and so no negative TCGA cases were included for testing. Identifiers for this data can be found at: <https://github.com/williamdee1/ThyCa-GAN/blob/main/data/ext_data_classification.csv>

The full NTE dataset is summarized in Table 5 of the main report.
