# Supplementary material for: Histopathological domain adaptation with generative adversarial networks: Bridging the domain gap between thyroid cancer histopathology datasets
Source: PLoS One. 2024 Dec 26;19(12):e0310417. doi: 10.1371/journal.pone.0310417 (PMC11670965; doi:10.1371/journal.pone.0310417)

**S3. Neoplastic region identification**

Supplementary material, Figure S2 shows two whole slide images after they have been annotated. From these identified regions of interest, 20 random image patches were extracted for each sample and used to form the NTE dataset.

**Supplementary Material, Figure S2.** Whole slide images where neoplastic regions of interest corresponding to the classification designation have been identified. (A) corresponds to the NIK-A036 NIFTP sample from Box A of the online Nikiforov repository. (B) relates to the TCGA Thyroid Carcinoma study FVPTC sample TCGA-EM-A4G1.


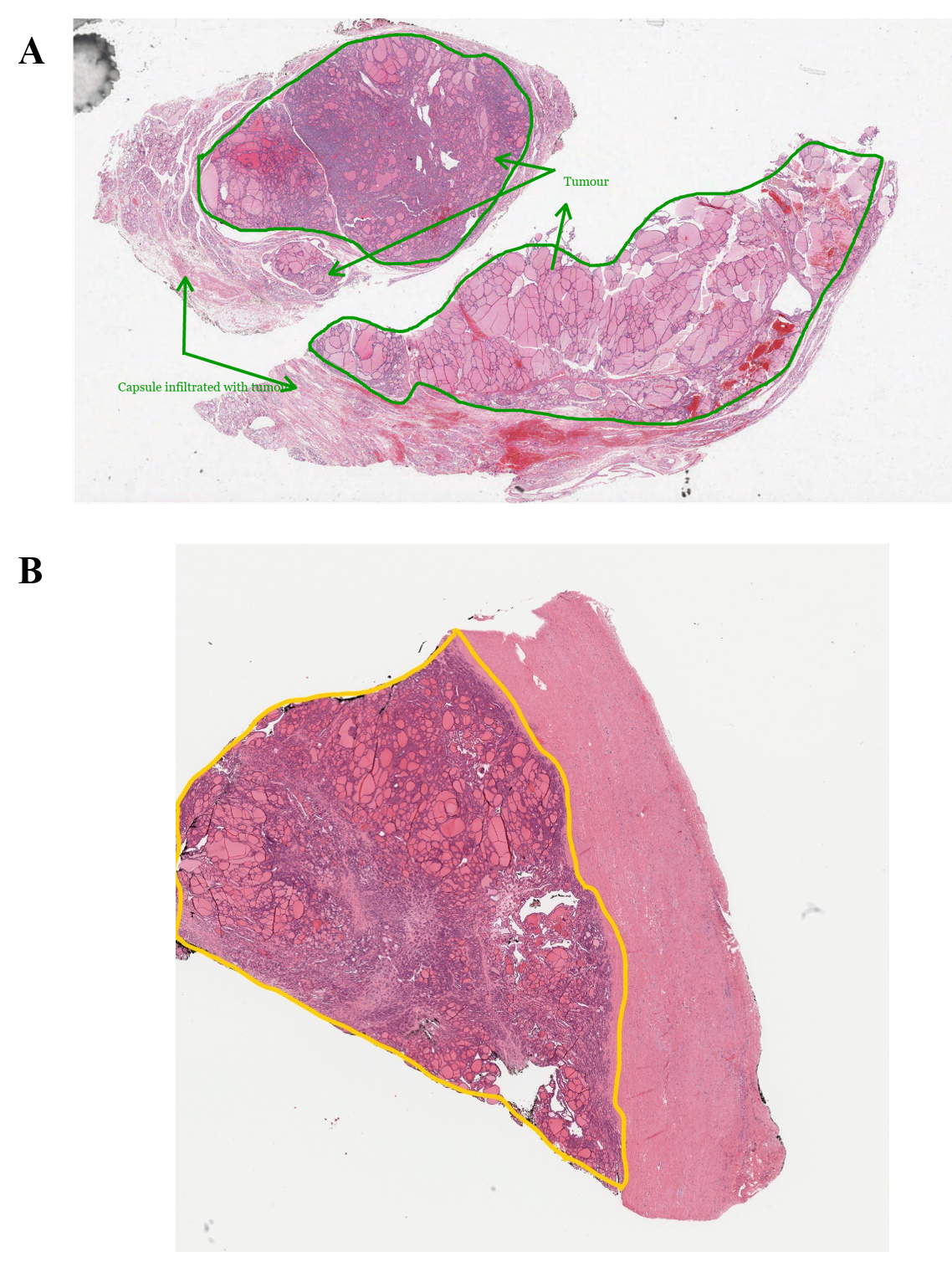

Supplement: S3 File — An overview of the selection of neoplastic regions from the downloaded WSIs. (DOCX) [file pone.0310417.s003.docx]
