# Supplementary material for: Histopathological domain adaptation with generative adversarial networks: Bridging the domain gap between thyroid cancer histopathology datasets
Source: PLoS One. 2024 Dec 26;19(12):e0310417. doi: 10.1371/journal.pone.0310417 (PMC11670965; doi:10.1371/journal.pone.0310417)
Supplement: S4 File — Tables displaying the amount of GAN-generated synthetic samples included during model training for each augmentation strategy included within the report. (DOCX) [file pone.0310417.s004.docx]

**S4. GAN augmentations**

This section includes details of the two GAN augmentation strategies – using either a GAN trained to produce binary-labelled or multi-class-labelled images. Supplementary material, Table S1 and Table S2 show the number of GAN synthetic images added to each class during model training according to the strategy selected.

**Supplementary Material, Table S1.** Table showing how many GAN-generated images are added to each classification type for each GAN augmentation strategy. In the case of cross-validated training, only 60% of the data is used for training the model, so the GAN augmentations are based off those 910 original real images. When using a binary-conditioned GAN the samples added are either designated to be PTC-like or non-PTC-like. If the GAN was trained conditionally with multi-class subtyping, then samples are added according to individual subtypes.

**Cross-Validated Training: Training Data = 60%**

| **Binary Class** | **No. Images** | **Binary Augmentation** |
| --- | --- | --- |
| PTC-like | 400 | 800 (+400) |
| Non-PTC-like | 510 | 1,020 (+510) |
|  | **910** | **1,820 (+910)** |
|  |  |  |
| **Multi Class** | **No. Images** | **Multi-Class Augmentation** |
| PTC | 310 | 640 (+330) |
| NIFTP | 44 | 640 (+596) |
| FVPTC | 46 | 640 (+594) |
| FA | 320 | 640 (+320) |
| FTC | 190 | 640 (+450) |
|  | **910** | **3,200 (+2,290)** |

**Supplementary Material, Table S2.** Table showing how many GAN-generated images are added to each classification type for each GAN augmentation strategy. When the model will be tested on the NTE data, 80% of the T&T data is used to train the model, therefore the GAN augmentations are based off those 1,213 original real images. When using a binary-conditioned GAN the samples added are either designated to be PTC-like or non-PTC-like. If the GAN was trained conditionally, then samples are added according to individual subtypes.

**Full Model Training: Training Data = 80%**

| **Binary Class** | **No. Images** | **Binary Augmentation** |
| --- | --- | --- |
| PTC-like | 533 | 1,066 (+533) |
| Non-PTC-like | 680 | 1,360 (+680) |
|  | **1,213** | **2,426 (+1,213)** |
|  |  |  |
| **Multi Class** | **No. Images** | **Multi-Class Augmentation** |
| PTC | 403 | 840 (+437) |
| NIFTP | 64 | 840 (+776) |
| FVPTC | 66 | 840 (+774) |
| FA | 420 | 840 (+420) |
| FTC | 260 | 840 (+580) |
|  | **1,213** | **4,200 (+2,987)** |
