# Supplementary material for: Histopathological domain adaptation with generative adversarial networks: Bridging the domain gap between thyroid cancer histopathology datasets
Source: PLoS One. 2024 Dec 26;19(12):e0310417. doi: 10.1371/journal.pone.0310417 (PMC11670965; doi:10.1371/journal.pone.0310417)
Supplement: Attachment — Submitted filename: comment for author histopathology.docx [file pone.0310417.s005.docx]

Histopathological domain adaptation with generative adversarial networks: Bridging the domain gap between thyroid cancer histopathology datasets

First of all, I want to thank you for giving me the chance to review this intriguing work  entitled “Histopathological domain adaptation with generative adversarial networks: Bridging the domain gap between thyroid cancer histopathology datasets”. In this manuscript, research on an important and pertinent subject is presented. It would be interesting to readers of this journal and provide them with pertinent information. After making some general observations, I will put specific topics under each section that have been suggested for modification.

**General Comments**

The methodology and results portions of the document must be made explicit before publication. For aid with fundamental grammar and sentence structure improvements, the authors could also benefit from the services of an editor.

PLOS ONE Comments to the Author
1. Is the manuscript technically sound, and do the data support the conclusions?
The manuscript must describe a technically sound piece of scientific research with data that supports the conclusions. Experiments must have been conducted rigorously, with appropriate controls, replication, and sample sizes. The conclusions must be drawn appropriately based on the data presented.

**Write in part**

2. Has the statistical analysis been performed appropriately and rigorously?

**Yes**

3. Have the authors made all data underlying the findings in their manuscript fully available?

**No**

4. Is the manuscript presented in an intelligible fashion and written in Standard English?

**Yes**

5. Review Comments to the Author

The draft is well-written. A native English speaker's edition or Journal English language services are necessary, though.

6. PLOS authors have the option to publish the peer review history of their article
Do you want your identity to be public for this peer review?

  Yes, Shemsu Oumer Ebro

**Introduction:** There are certain points in the introduction that want modification. The authors' aim to bridge the gap in the literature will be briefly presented to readers. Additionally, please edit and revise the first section, removing as many terms as possible..

**Methods:** part describing the method needs to be changed more thoroughly. In particular, the measuring section needs to be rewritten and condensed for more powerful clarity. It is necessary to talk about the instrument's validity and reliability as well as whether the questionnaire was utilized or modified for purposes other than collecting data.

**Conclusion:** There's an opportunity for doubt in your conclusion. It would be better if you wrote it using the information you discovered.

**References**
Kindly review and revise your references in compliance with PLOS policies.

Since PLOS ONE does not copyedit submitted submissions, it is advised that authors hire an editor to assist with any unclear or grammatical issues that arise throughout the text.

A few standardised grammatical and phrase construction changes are required before publication.
