# Supplementary material for: Histopathological domain adaptation with generative adversarial networks: Bridging the domain gap between thyroid cancer histopathology datasets
Source: PLoS One. 2024 Dec 26;19(12):e0310417. doi: 10.1371/journal.pone.0310417 (PMC11670965; doi:10.1371/journal.pone.0310417)
Supplement: Attachment — Submitted filename: Response to Reviewers.docx [file pone.0310417.s006.docx]

**PLOS ONE – Response to Reviewer Comments: Histopathological domain adaptation with generative adversarial networks: Bridging the domain gap between thyroid cancer histopathology datasets**

**Reviewer 1:**

*Comment 1:*

Introduction: There are certain points in the introduction that want modification. The authors' aim to bridge the gap in the literature will be briefly presented to readers. Additionally, please edit and revise the first section, removing as many terms as possible.

*Author response:* We have now presented an outline of the domain gap problem within the introduction, it’s presence within the data used for this research and the incentive to use a Generative model to overcome it, please see the wording below (blue words are additions to the text):

The authors suggested this failure to generalize was due to a lack of diverse training data, a common problem when applying machine learning methods to (often small) medical imaging datasets [14]. This issue can be exacerbated when using deep learning, which typically requires large datasets to produce highly generalizable models [28]. A lack of training data diversity can be especially problematic when there is a large domain gap present between datasets, i.e., differences in data distributions and/or feature representations caused by the underlying processes behind gathering and processing the data. These domain differences can obscure the underlying biological information, making it difficult to train robust models which can adapt well to the new data.

A Uniform Manifold Approximation and Projection (UMAP) [30] plot can be used to visualize the domain gap between datasets. Images from the Tharun and Thompson (T&T) [20] and Nikiforov datasets [31] were passed through a pretrained ResNet50 [29] model to obtain embeddings, before being represented in a two-dimensional latent space using UMAP (Fig 1). The separation between the two sets of embeddings demonstrates that the data distributions are clearly distinct from each other – i.e., there are stronger differences caused by the domain origination, than similarities relating to samples which share the same histopathological classifications.

One potential solution to bridge the domain gap is to use generative adversarial networks (GANs). GANs were introduced by Goodfellow et al. [32] as a method of producing high quality synthetic (fake) images which approximate an underlying real data distribution. Adversarial training methods have previously been applied successfully to generate artificial data in MRI reconstruction and tumour segmentation [33], X-ray organ segmentation [34, 35], and virtual slide staining [36–38].

**Fig 1:** UMAP visualization of the domain gap between the T&T [20] and the Nikiforov datasets [31]. Images from both datasets were passed through a pretrained ResNet50 [29] model to obtain embedding representations. The figure shows a clear separation between the two datasets along the first UMAP dimension. This gap can have a negative impact on the ability of a model trained on one dataset being able to generalize to predict the other.

*Comment 2:*

Methods: part describing the method needs to be changed more thoroughly. In particular, the measuring section needs to be rewritten and condensed for more powerful clarity. It is necessary to talk about the instrument's validity and reliability as well as whether the questionnaire was utilized or modified for purposes other than collecting data.

*Author response:* Thank you, we have gone through and amended the Methods section to be more concise. There was no questionnaire used in this research and we acquired histopathology data from external papers so the measuring instrument information is not available to us but could be requested from the original paper authors if required by any future researchers. None of the original papers suggested a lack of validity or reliability in terms of the diagnosis or imaging quality, unless already discussed in the text, i.e. the difficulty of assigning diagnoses to minority subtypes with subjective classification criteria.

*Comment 3:*

Conclusion: There's an opportunity for doubt in your conclusion. It would be better if you wrote it using the information you discovered.

*Author response: We have re-written the discussion section entirely based on the new results and removed doubt from our conclusions regarding the GANs ability to improve model robustness when generalizing to unseen data.*

Comment 4:

References: Kindly review and revise your references in compliance with PLOS policies. Since PLOS ONE does not copyedit submitted submissions, it is advised that authors hire an editor to assist with any unclear or grammatical issues that arise throughout the text. A few standardised grammatical and phrase construction changes are required before publication.

*Author response:* Thank you for your suggestion, we have re-reviewed the wording and phrasing throughout and sent the draft to be looked over by an editor prior to resubmitting.

**Reviewer 2:**

*Comment 1:*

Beyond the descriptions reported on S1, I remain curious to know whether for subtypes detection further genotype/phenotype data integration can be combined into the approach to validate or robustify the observed improvements. Are the authors considering practical steps in such directions? If so, can they add comments to their discussion part?

*Author response:* Thank you for the interesting suggestion, we are not considering steps in this direction as we believe it’s out of the scope of this research, though it is a great proposal for future research. We believe the main purpose of this research is to evaluate the efficacy of using a GAN to augment data sufficiently to improve classifier performance on minority classes where there is a lack of training data. Fusing additional data modalities, while interesting and definitely worth exploring in future research, would not validate or make more robust the improvements demonstrated in this paper related to the GAN data augmentation. We believe integrating such data here would make the purpose of this research less clear but remains a potential area for future studies that have access to other aligned data resources.

**Reviewer 3:**

*Comment 1:*

Improvement of image quality in Figures. It might be better to move Fig 5 and Tables 4-5 to the supplementary material due to redundancy. It would be preferable for Fig 6 to appear earlier, from the method section to the results. The quality of the figures needs to be improved.

*Author response: We have improved image quality across all figures, re-running all figure generation and using photo-editing software to ensure the figures are high resolution.*

*Comment 2:*

Domain gap bridging. In Fig 1, consider adding a figure that better illustrates the bridging effect. Currently, the figure seems to only show batch effects, making it less meaningful.

*Author response: The domain gap refers to the difference in underlying data distributions between two or more datasets. In this case it has arisen due to the data being collected in different environments (hospitals), but there are also numerous batch effects present in the data due to the experimental conditions and data collection procedures.*

*We have removed this figure, which was originally from the reference paper by Bohland et al. In it’s place we have created our own figure, which has much higher resolution, by passing the images from the T&T and Nikiforov datasets through a Resnet50 classifier and then visualizing the embeddings using Uniform Manifold Approximation and Projection (UMAP). This effectively mirrors what the original authors performed. However, now we have explicitly labelled the domain gap that is present between the datasets, shown as the separation of the datapoints along the UMAP1 axis. See the new figure and annotation in response to reviewer 1, comment 1.*

*Comment 3:*

Performance on Niki-TCGA dataset. The final performance on the Niki-TCGA dataset is quite poor, which may indicate limited generalizability. What could be the reason for the low prediction performance on the Niki-TCGA set even for PTC-like cases? Could there be a difference in performance depending on the number of GAN-generated images?

*Author response: We agreed that the prior performance on the Niki-TCGA (now NTE) dataset was relatively poor. To combat this, we altered the structure of the model architecture, incorporating the transformer-based SwinV2 model architecture in place of the ResNet101 architecture. This, combined with the GAN augmentation, improved results significantly. Additionally, we decided that accuracy in the case of the external test data was a poor metric to use due to the class imbalance, and so we evaluated the results using precision, recall and AUC scores instead.*

*Comment 4:*

Sharing GAN-generated images. It could be beneficial to share the GAN-generated images along with their FID scores to provide a clearer understanding of the image quality and relevance.

*Author response: The GAN models have been shared on the GitHub page, with instructions for how to generate images from them, so anyone can use them and, if they use the seeds we have provided, will be able to replicate the generated images used in this research.*

*Comment 5:*

F1 scores for MC model. For the MC model, it would be useful to present the F1 scores for each class individually to provide a more detailed performance evaluation.

*Author response: We have changed the evaluation criteria based on the incorporation of a third dataset into the external test data. Now precision, recall and AUC form the evaluation basis which is much more appropriate considering the class imbalance. F1 scores for each class in this context would likely not be appropriate given we are classifying each sample into a binary designation (PTC-like or non-PTC-like) rather than this being a multi-class classification problem.*

**Reviewer 4:**

*Comment 1:*

Suggesting a mention of the S5 figure in the creation of the Nikki-TCGA dataset to improve understanding. Additionally, ensure that the upper and lower figures in S5 have the same UMAP positions for better clarity.

*Author response: We have removed the S5 figure and created a new graphic which includes the additional domain data (from Eftimie et al.’s [CITE] research) that we have added since we completed revisions after this feedback. We have ensured all plots within this new figure have the same UMAP axes, see below:*

**Fig 5:** Images from the T&T and NTE dataset were passed through a pre-trained ResNet101 classifier to produce embeddings. These embeddings were then visualized using the Uniform Manifold Approximation (UMAP) and coloured according to: (A) dataset domain, referring to the paper which produced and shared the original images; (B) classification as having PTC-like or non-PTC-like nuclei present; (C) subtype diagnosis. As can be seen in (A) the Eftimie FA samples are more similar to the T&T data, as represented by their closeness in two-dimensional space, than the Nikiforov or TCGA samples are, models with therefore likely generalize better from the T&T data to the Eftimie data than they will to the Nikiforov or TCGA data.

*Comment 2:* Recommend providing an explanation of Formula 1 and detail the terms used to enhance reader comprehension.

*Author response: Thank you for the suggestion. There are numerous papers, many of which are linked in this research, which go into detail about how GANs function – we agree that this is not necessary in this paper at the cost of reader comprehension and so we have removed the formula entirely and simplified the language used to describe the process. See below for the new main text (in blue):*

… GANs are comprised of two neural network models, referred to as the generator and the discriminator. The generator aims to learn to approximate the underlying distribution of the training data to generate high-quality synthetic images. The discriminator conversely learns to discern the difference between these GAN-generated fakes and the real images.

The two networks compete during training to improve at their respective roles, producing realistic synthetic examples and detecting fake images. An equilibrium is reached when the generator is producing images which are indistinguishable from the underlying real data, and thus the discriminator can make predictions about whether an image is real or fake with only 50% accuracy.

*Comment 3:* Suggest considering more robust metrics than FID to evaluate the model’s performance.

*Author response: We also agree that FID is not the ideal metric to evaluate the quality of synthetic GAN images. In the main text we have mentioned that other papers have signalled that FID can’t be overly relied upon (see below). We have used it in this paper to provide some context behind the success of the GAN training compared to successful GANs in other domains – hence the comparison with the original StyleGan2 paper FID scores on larger datasets. However, that is the purpose of using these synthetic images to train a model to generalize better to unseen data, produced in a different domain – providing a litmus test of their ability to be beneficial to a model by increasing the diversity of training data when the original data is relatively scarce. We have written this in the main text (blue text):*

A lower FID score implies a greater alignment between the fake and real images and has been correlated with human judgement regarding the quality of the generated images [49]. Despite this, the metric is not considered perfect, and several papers have warned against over-reliance on FID to assess GAN improvement [45, 49].

In this paper FID was used as the target metric for GAN training. Despite this, the true assessment of the quality of the generated images will be whether their addition to the real images during training improves the classification performance of the deep learning model on unseen test data. This report therefore assesses the impact of GANs in an applied scenario, rather than purely evaluating the generative technique in isolation.

*Additionally, we have also changed the evaluation criteria for judging how well the models trained using synthetic data generalize to the external domain data – see Reviewer 3 Comment 5 response for more detail.*

*Comment 4:*

Provide an explanation of the differences on pictures from the datasets. And lack of explanation on the differences between datasets.

*Author response: We have added detail to the explanation of the datasets within the Methods: NTE dataset section and added a figure showing the differences between the images across the different dataset domains.*

*Comment 5:*

Consider explaining why the 100 value was chosen on the data augmentation, and not any other potential number.

*Author response: We explored several different augmentation strategies during our work, not just the two presented in the paper. However, the purpose of this research was to show how additional synthetic samples impact a model’s ability to generalize to unseen data, rather than being an exhaustive exploration to find the optimal augmentation number to increase samples by – though this could be a worthwhile area for future research.*

*We believe that an 100% increase in binary labelled samples (in the case of the binary GAN) or the equalization in the number of samples across classes (in the case of the multi-class GAN) are the most succinct two approaches for a reader to digest and for us to discuss conclusions from regarding our initial aims. We have added some additional commentary into the Methods and Discussion sections relating to the augmentations.*

*Comment 6:*

Explore the possibility of connecting GANs to improve performance.

*Author response: This is an interesting suggestion and connecting GANs could improve performance, however we consider doing this within this research to be beyond the scope. Despite this we have added the following to the discussion, highlighting a potential future area for further investigation:*

**Editor:**

*Comment 1:*

In addition, for the general readers, and young researcher in this thyroid cancer field, it would be interesting to add some molecular background information in the introduction.
Particularly, these seminal articles on BRAF, TERT, RAS, ALK, mTOR, etc., and other can be cited (PMID: 27387551; PMID: 23766237; PMID: 25024077; PMID: 21596819; PMID: 30918329).

*Author response:* Thank you for suggesting and providing links to the articles above, we have incorporated some of this information into the introduction now, see wording below:

… Notably, several genetic mutations have been implicated in the pathogenesis of PTC especially BRAF V600E (Murugan at al., 2016). The ALK gene mutation has been observed in various thyroid cancers, including PTC, follicular thyroid carcinoma (FTC), and undifferentiated anaplastic thyroid cancer (Murugan and Xing et al., 2011). Additionally, the C228T promoter mutation in the TERT gene is associated with the BRAF V600E mutation in PTC (Liu et al., 2013; Xing et al., 2014).

Other genetic mutations reported in various thyroid cancer include RAS (Younis, 2017; Nikiforov, 2008), PIK3CA, AKT1, PTEN (Prete et al, 2020), mTOR (Murugan et al., 2019), and chromosomal rearrangements involving RET/PTC and PAX8-PPARɣ genes (Nikiforov, 2008). These genetic mutations can deregulate the mitogen-activated protein kinase (MAPK) and phosphatidylinositol-3 kinase (PI3K)/AKT signalling pathways that is crucial to the pathogenesis of thyroid cancer (Younis, 2017). It is noteworthy to mention that MAPK activation is crucial for the initiation of PTC (Prete et al, 2020). Other signalling pathways include p53 and Wnt/β-catenin (Prete et al, 2020).

**Original Copy of Reviewer Comments Received 7^th^ February 2024:**

The study by William et al., is interesting and this was also reflected in reviewers' comments.

However, the reviewers raise some important concerns and those points to be addressed before considering it for publication.

In addition, for the general readers, and young researcher in this thyroid cancer field, it would be interesting to add some molecular background information in the introduction.
Particularly, these seminal articles on BRAF, TERT, RAS, ALK, mTOR, etc., and other can be cited (PMID: 27387551; PMID: 23766237; PMID: 25024077; PMID: 21596819; PMID: 30918329).


[Note: HTML markup is below. Please do not edit.]

Reviewers' comments:

Reviewer's Responses to Questions

**Comments to the Author**

1. Is the manuscript technically sound, and do the data support the conclusions?

The manuscript must describe a technically sound piece of scientific research with data that supports the conclusions. Experiments must have been conducted rigorously, with appropriate controls, replication, and sample sizes. The conclusions must be drawn appropriately based on the data presented.

Reviewer #1: Yes

Reviewer #2: Yes

Reviewer #3: Yes

Reviewer #4: Yes

2. Has the statistical analysis been performed appropriately and rigorously?

Reviewer #1: No

Reviewer #2: Yes

Reviewer #3: Yes

Reviewer #4: Yes

3. Have the authors made all data underlying the findings in their manuscript fully available?

The [PLOS Data policy](https://eur01.safelinks.protection.outlook.com/?url=http%3A%2F%2Fwww.plosone.org%2Fstatic%2Fpolicies.action%23sharing&data=05%7C02%7Cw.t.dee%40qmul.ac.uk%7C2199260eac21489b5fa308dc280f5656%7C569df091b01340e386eebd9cb9e25814%7C0%7C0%7C638429293686844493%7CUnknown%7CTWFpbGZsb3d8eyJWIjoiMC4wLjAwMDAiLCJQIjoiV2luMzIiLCJBTiI6Ik1haWwiLCJXVCI6Mn0%3D%7C0%7C%7C%7C&sdata=Q6q2orOpS5HUvh477LgTXvGkCC%2BoukUvkcrCt1z4P20%3D&reserved=0) requires authors to make all data underlying the findings described in their manuscript fully available without restriction, with rare exception (please refer to the Data Availability Statement in the manuscript PDF file). The data should be provided as part of the manuscript or its supporting information, or deposited to a public repository. For example, in addition to summary statistics, the data points behind means, medians and variance measures should be available. If there are restrictions on publicly sharing data—e.g. participant privacy or use of data from a third party—those must be specified.

Reviewer #1: Yes

Reviewer #2: Yes

Reviewer #3: No

Reviewer #4: Yes

4. Is the manuscript presented in an intelligible fashion and written in standard English?

PLOS ONE does not copyedit accepted manuscripts, so the language in submitted articles must be clear, correct, and unambiguous. Any typographical or grammatical errors should be corrected at revision, so please note any specific errors here.

Reviewer #1: Yes

Reviewer #2: Yes

Reviewer #3: Yes

Reviewer #4: Yes

5. Review Comments to the Author

Please use the space provided to explain your answers to the questions above. You may also include additional comments for the author, including concerns about dual publication, research ethics, or publication ethics. (Please upload your review as an attachment if it exceeds 20,000 characters)

Reviewer #1: It would be better if you wrote it using the information you discovered.
methodology part and result parts that authors hire an editor to assist with any unclear or grammatical issues that arise throughout the text.
A few standardised grammatical and phrase construction changes are required before publication.

*Additional Reviewer 1 Comments from Attached Review Document:*

Introduction: There are certain points in the introduction that want modification. The authors' aim to bridge the gap in the literature will be briefly presented to readers. Additionally, please edit and revise the first section, removing as many terms as possible.

Methods: part describing the method needs to be changed more thoroughly. In particular, the measuring section needs to be rewritten and condensed for more powerful clarity. It is necessary to talk about the instrument's validity and reliability as well as whether the questionnaire was utilized or modified for purposes other than collecting data.

Conclusion: There's an opportunity for doubt in your conclusion. It would be better if you wrote it using the information you discovered.

References: Kindly review and revise your references in compliance with PLOS policies. Since PLOS ONE does not copyedit submitted submissions, it is advised that authors hire an editor to assist with any unclear or grammatical issues that arise throughout the text. A few standardised grammatical and phrase construction changes are required before publication.

Reviewer #2: good job, the paper presents an important problem and a relevant methodological approach within a line of research that offers at times reason for discussion in terms of explainability more than efficacy or effectiveness.
Beyond the descriptions reported on S1, I remain curious to know whether for subtypes detection further genotype/phenotype data integration can be combined into the approach to validate or robustify the observed improvements. Are the authors considering practical steps in such directions? If so, can they add comments to their discussion part?

Reviewer #3: The manuscript presents a comprehensive study on the use of Generative Adversarial Networks (GANs) to generate synthetic thyroid histopathology images to augment a limited dataset for improving the generalizability of deep learning classifiers (DLCs) in medical imaging. The key focus is on addressing the challenges of data scarcity and heterogeneity in thyroid cancer diagnosis, particularly differentiated thyroid cancer (DTC) and its most common subtype, papillary thyroid carcinoma (PTC), along with more challenging minority subtypes like NIFTP and FVPTC.

1. Improvement of image quality in Figures
• It might be better to move Fig 5 and Tables 4-5 to the supplementary material due to redundancy.
• It would be preferable for Fig 6 to appear earlier, from the method section to the results.
• The quality of the figures needs to be improved.
2. Domain gap bridging
• In Fig 1, consider adding a figure that better illustrates the bridging effect. Currently, the figure seems to only show batch effects, making it less meaningful.
3. Performance on Niki-TCGA dataset
• The final performance on the Niki-TCGA dataset is quite poor, which may indicate limited generalizability. What could be the reason for the low prediction performance on the Niki-TCGA set even for PTC-like cases?. Could there be a difference in performance depending on the number of GAN-generated images?
4. Sharing GAN-generated images
• It could be beneficial to share the GAN-generated images along with their FID scores to provide a clearer understanding of the image quality and relevance.
5. F1 scores for MC model
• For the MC model, it would be useful to present the F1 scores for each class individually to provide a more detailed performance evaluation.
 
Overall, the manuscript presents a promising approach to leveraging advanced machine learning techniques to improve the accuracy and generalizability of diagnostic models in thyroid cancer histopathology, with potential for impacting clinical practice.

Reviewer #4: Suggesting a mention of the S5 figure in the creation of the Nikki-TCGA dataset to improve understanding. Additionally, ensure that the upper and lower figures in S5 have the same UMAP positions for better clarity.

Recommend providing an explanation of Formula 1 and detail the terms used to enhance reader comprehension

Suggest considering more robust metrics than FID to evaluate the model’s performance.

Provide an explanation of the differences on pictures from the datasets. And lack of explanation on the differences between datasets.

Consider explaining why the 100 value was chosen on the data augmentation, and not any other potential number.

Explore the possibility of connecting GANs to improve performance.

6. PLOS authors have the option to publish the peer review history of their article ([what does this mean?](https://eur01.safelinks.protection.outlook.com/?url=https%3A%2F%2Fjournals.plos.org%2Fplosone%2Fs%2Feditorial-and-peer-review-process%23loc-peer-review-history&data=05%7C02%7Cw.t.dee%40qmul.ac.uk%7C2199260eac21489b5fa308dc280f5656%7C569df091b01340e386eebd9cb9e25814%7C0%7C0%7C638429293686851464%7CUnknown%7CTWFpbGZsb3d8eyJWIjoiMC4wLjAwMDAiLCJQIjoiV2luMzIiLCJBTiI6Ik1haWwiLCJXVCI6Mn0%3D%7C0%7C%7C%7C&sdata=gfo7vpEXXRvoKWcXogdKzlzN%2FOIjR5f8BSoXbaEUMNM%3D&reserved=0)). If published, this will include your full peer review and any attached files.


If you choose “no”, your identity will remain anonymous but your review may still be made public.


**Do you want your identity to be public for this peer review?** For information about this choice, including consent withdrawal, please see our [Privacy Policy](https://eur01.safelinks.protection.outlook.com/?url=https%3A%2F%2Fwww.plos.org%2Fprivacy-policy&data=05%7C02%7Cw.t.dee%40qmul.ac.uk%7C2199260eac21489b5fa308dc280f5656%7C569df091b01340e386eebd9cb9e25814%7C0%7C0%7C638429293686858931%7CUnknown%7CTWFpbGZsb3d8eyJWIjoiMC4wLjAwMDAiLCJQIjoiV2luMzIiLCJBTiI6Ik1haWwiLCJXVCI6Mn0%3D%7C0%7C%7C%7C&sdata=s5opyKc%2BV8WgQ6j0wIj4HuXhkyPgEj1XnexJkjNh%2FBI%3D&reserved=0).

Reviewer #1: **Yes:** Shemsu Oumer Ebro

Reviewer #2: **Yes:** Enrico Capobianco

Reviewer #3: No

Reviewer #4: No
